# Cardiovascular Magnetic Resonance Imaging Evidence of Edema in Chronic Chagasic Cardiomyopathy

**DOI:** 10.1155/2019/6420364

**Published:** 2019-09-05

**Authors:** Andrés Diaz, Juan José Diaztagle, Alejandro Olaya, Guillermo Mora, Ignacio López-Lima, Carolina Ayala, Gina P. P. Infante, Néstor Galizio, Frida T. Manrique, Julian F. Forero, Hector M. Medina, Josep Brugada

**Affiliations:** ^1^Hospital San José, Bogotá, Colombia; ^2^Fundación Universitaria de Ciencias de la Salud, Bogotá, Colombia; ^3^Escola de Doctorat, Universitat de Barcelona, Barcelona, Spain; ^4^Facultad de Medicina, Universidad Nacional de Colombia, Bogotá, Colombia; ^5^Fundación Santa Fe de Bogotá, Bogotá, Colombia; ^6^Hospital Dr. Guillermo Rawson, San Juan, Argentina; ^7^Statistical Analysis and Research Consulting, Bogotá, Colombia; ^8^Hospital Universitario Fundación Favaloro, Buenos Aires, Argentina; ^9^Departamento de Imagen Diagnóstica, Fundación Cardioinfantil, Bogotá, Colombia; ^10^Hospital Clínic de Barcelona, Barcelona, Spain

## Abstract

The persistence of inflammatory processes in the myocardium in varying degrees of chronic Chagas heart disease has been poorly investigated. We hypothesized that edema could occur in patients with chronic chagasic cardiomyopathy and corresponds to the persistence of inflammatory processes in the myocardium. Eighty-two Chagas disease (CD) seropositive patients (64.6% females; age = 58.9 ± 9.9) without ischemic heart disease or conditions that cause myocardial fibrosis and dilation were considered. Late gadolinium enhancement (LGE) and T2-weighted magnetic resonance imaging of edema were obtained and represented using a 17-segment model. Patients were divided into three clinical groups according to the left ventricular (LV) ejection fraction (EF) as G1 (EF > 60%; *n*=37), G2 (35% > EF < 60%; *n*=33), and G3 (EF < 35%; *n*=12). Comparisons were performed by the Fisher or ANOVA tests. Bonferroni post hoc, Spearman correlation, and multiple correspondence analyses were also performed. Edema was observed in 8 (9.8%) patients; 2 (5.4%) of G1, 4 (12.1%) of G2, and 2 (16.7%) of G3. It was observed at the basal inferolateral segment in 7 (87.5%) cases. LGE was observed in 48 (58.5%) patients; 16 (43.2%) of G1, 21 (63.6%) of G2, and 11 (91.7%) of G3 (*p* < 0.05). It was observed in the basal inferior/inferolateral/anterolateral segments in 35 (72.9%) patients and in the apical anterior/inferior/lateral and apex segments in 21 (43.7%), with midwall (85.4%; *n*=41), subendocardial (56.3%; *n*=27), subepicardial (54.2%; *n*=26), transmural (31.2%; *n*=15), and RV (1.2%; *n*=1) distribution. Subendocardial lesions were observed only in patients with LVEF < 35%. There was no involvement of the mid-inferolateral/anterolateral segments with an LVEF > 35% (*p* < 0.05). Deteriorations of the LV and RV systolic functions were positively correlated (*r*_*s*_=0.69; *p* < 0.05) without evidence of LGE in the RV. Edema can be found in patients with chagasic cardiomyopathy in the chronic stage. In later stages of cardiac dilation with low LVEF, the LGE pattern involves subendocardium and mid locations. Deteriorations of RV and LV are positively correlated without evidence of fibrosis in the RV.

## 1. Introduction

Chagasic heart disease, caused by *Trypanosoma cruzi* infection, is a pathology of crucial interest in public health. The vectors, bug members of the subfamily Triatominae, are hematophagous insects distributed mainly from the southern United States to South America [1]. It is estimated that 8 to 10 million people are infected in the world [2]. In the Americas, the incidence is approximately 41,200 new cases per year and it is estimated that 108,595,000 people are at risk [3]. Additionally, in this continent, almost two million women in reproductive age are infected, of which 4 to 8% would transmit the infection to the fetus [4]. In Colombia, between 700,000 and 1,200,000 inhabitants are infected and 8,000,000 individuals are at risk of acquiring the infection [5].

After infection with *T. cruzi*, the asymptomatic phase can last for decades until unknown triggers initiate cardiac involvement in >90% of the cases [6], which is mainly characterized by chronic myocarditis that involves interstitial and diffuse fibrosis [7]. This is a crucial clinical aspect of Chagas disease because of its poor prognosis [8–10]. In fact, in a Brazilian hospital-based cohort of 1220 outpatients with heart failure of different etiologies, Chagas heart disease was the main prognostic factor for mortality [9]. Typically, death occurs in men between 33 and 72 years, affecting mainly vulnerable populations [11].

Recently, cardiac magnetic resonance (CMR) has been considered a sensitive technique to detect myocardial damage in Chagas heart disease [6, 12–14]; it is suitable to detect segmental wall motion abnormalities, particularly myocardial necrosis or fibrosis using the late gadolinium enhancement (LGE) technique [13–18] and heralds an adverse prognosis in nonischemic cardiomyopathy [19]. Grothues et al. reported CMR as the best method to assess ventricular function with an excellent interstudy reproducibility superior to two-dimensional echocardiography in normal, dilated, and hypertrophic hearts. In addition, CMR has demonstrated to detect myocardial edema using T2-weighted myocardial early gadolinium enhancement image sequences [20–22]. Nonetheless, this method has been scarcely used in the study of Chagas heart disease, and the findings that can guide the treatment and prognosis of those patients are unknown, especially in countries of greater risk and vulnerability. In this way, recognition of the CMR pattern of Chagas heart disease would be useful to detect myocardial involvement of patients in different clinical phases of this disease. Consequently, decisions regarding therapy, including heart transplantation, can be supported by CMR findings and in the knowledge of the long-term prognosis of affected patients [9, 14, 18]. Thus, the aim of this study is to characterize CMR findings in Chagas-seropositive patients and to confirm the hypothesis that edema could occur in patients with chronic chagasic cardiomyopathy and corresponds to the persistence of the inflammatory process in the myocardium.

## 2. Materials and Methods

This study was performed with 82 patients in different clinical phases of Chagas heart disease. Patients were divided into groups according to the left ventricular (LV) ejection fraction (EF) as G1 (EF > 60%; *n*=37), G2 (35% < EF > 60%; *n*=33), and G3 (EF < 35%; *n*=12). Sociodemographic data, clinical history, and comorbidities were obtained from patients' medical records. The inclusion criteria were patients over 18 years old, positive for Chagas disease according to the CDC criteria, epidemiological background, and two positive laboratory tests (immunoenzymatic assay tests-ELISA, and indirect immunofluorescence-IFI). Each patient signed a consent form to be part of the study.

The exclusion criteria considered refusal of the patient or their relatives to participate in the study diagnosis of coronary disease; dilated cardiomyopathy of ischemic origin and/or significant valvular disease; contraindications to magnetic resonance; presence of tattoos, metallic pigments, nonremovable metal prostheses, metallic sutures and/or heart devices not compatible with magnetic resonance; pregnancy; fever; severe claustrophobia; severe psychiatric disorders; creatinine <30 mL/minute in a 24-hour depuration test; and contraindication to gadolinium as a contrast medium.

Patients were recruited from the cardiology, electrophysiology, emergency, and hospitalization services of seven cardiovascular reference hospitals of the city of Bogotá DC, Colombia.

### 2.1. Magnetic Resonance Imaging Methods

CMR was performed in all selected patients using a Philips Ingenia 1.5 T scanner. The exploration protocol comprised an initial morphological evaluation considering bright and black-blood gradient-echo sequences in the axial and coronal planes. The functional evaluation considered maps of T1 and T2 sequences of cine resonance with the technique of steady-state free precession (SSFP). Additionally, a quantitative functional evaluation was carried out. Structural measures (diastolic and systolic diameter of the left ventricle, diastolic thickness of the walls, anterior systolic left atrial diameter, volume of the left atrium in two dimensions, mean lateral diameter of the right ventricle, and presence of pericardial effusion) and functional measures (ejection fraction of the left ventricle, estimation of pulmonary arterial pressure, and presence and degrees of mitral and tricuspid regurgitation) were obtained from each patient. Tissue characterization evaluated the presence of myocardial edema by means of T2 sequences with fat suppression and the presence of LGE with T1 IR acquisitions in 2D and 3D. Additionally, images of LGE were obtained specifying the location and degree of transmurality. The intravenous contrast material used was Gadobutrol (0.1 mmol/kg), a gadolinium-based MRI contrast agent.

The interpretation of the resonances was carried out by specialists in radiology and diagnostic images of the participating institutions. A second reading of the study was made by an expert in advanced cardiac imaging with experience in cardiovascular magnetic resonance analysis; the specialists were blinded with respect to the previous analysis, in order to estimate the reproducibility of the imaging variables obtained in the study.

### 2.2. Statistical Analysis

Comparisons of normally distributed continuous variables between groups were performed by the one-way analysis of variance (ANOVA) with the Bonferroni post hoc test for multiple comparisons. The nonparametric test for discrete variables and nonnormal continuous variables was the Kruskal–Wallis test by ranks. Normality was determined by the Shapiro–Wilk test. The Fisher exact test was used for proportion comparisons between the groups. A confidence level of 95% was used to considered test results statistically significant.

Multiple correspondence analysis (MCA), a multivariate data analysis, was employed to construct relationships among categorical variables associated with the presence and location of LGE in the different LVEF groups. Chi-squared tests were performed to verify the association between categorical variables. All statistical analyses were performed using the R language [23].

## 3. Results and Discussion

Table 1 summarizes clinical characteristics for each group according to the considered variables. The median age was 58.9 years (ranging from 33 to 80), 64.6% were women, and the mean body mass index was 26.2 (SD 4.35). Some of these patients suffered from high blood pressure (47.6%), diabetes mellitus (8.9%), and dyslipidemia (15.4%) or were smokers (3.8%). Those patients were classified in the NYHA functional class I (54.9%: *n*=45), class II (43.9%; *n*=36), and class III (1.2%; *n*=1).

**Figure 1:**
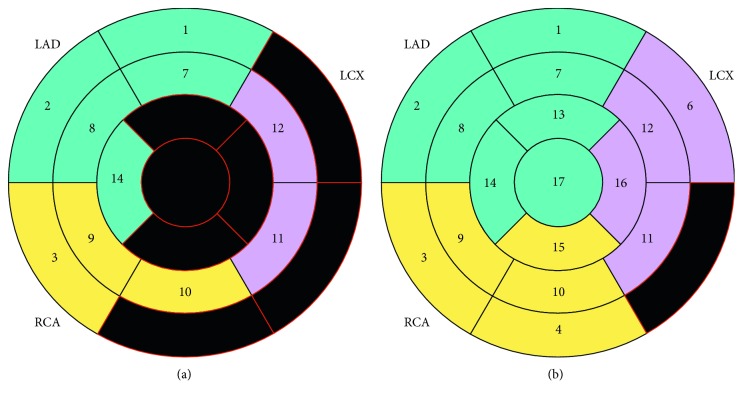
Results of the (a) LGE and (b) edema distribution in a 17-segment heart model. LAD: left anterior descending; RCA: right coronary artery; LCX: left circumflex. Modified from http://www.pmod.com/files/download/v34/doc/pcardp/3615.htm.

**Figure 2:**
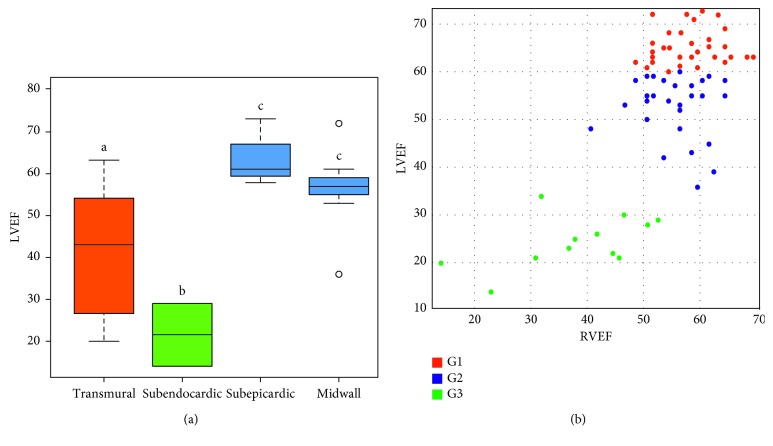
(a) LVEF of different LGE distributions and (b) scatterplot of LVEF and RVEF between groups (*r*_*s*_=0.69).

LGE was observed in 48 (58.5%) patients; 16 (43.2%) of G1, 21 (63.6%) of G2, and 11 (91.7%) of G3 (*p* < 0.05). It was observed in the basal inferior/inferolateral/anterolateral segments in 35 (72.9%) patients and in the apical anterior/inferior/lateral and apex segments in 21 (43.7%) patients, with midwall (85.4%; *n*=41), subendocardial (56.2%; *n*=27), subepicardial (54.2%; *n*=26), transmural (31.2%; *n*=15), and RV (1.2%; *n*=1) distribution. The distribution of LGE in a 17-segment model (AHA) is shown in Figure 1(a). Subendocardial lesions were more frequently observed in patients with LVEF <35% (*p* < 0.05) (Figure 2(a)). Those patients also presented a greater involvement of the apical and mesial segments (*p* < 0.05). There was no involvement of the midwall and subepicardial segments with an LVEF >35% (*p* < 0.05) (Figure 2(a)). G3 patients showed a significantly higher LV telediastolic volume (*p* < 0.01), LV telediastolic volume index (*p* < 0.01), LV telesystolic volume (*p* < 0.01), LV telesystolic volume index (*p* < 0.01), LV mass (*p* < 0.01), LV mass index (*p* < 0.01), VD telesystolic volume (*p* < 0.01), and VD telesystolic volume index (*p* < 0.01) when compared to the other groups. There were no differences in the VD telediastolic volumes and VD telediastolic indexes between the groups. This predominant distribution at the inferolateral wall of the LV and a heterogeneous distribution inside the myocardium is in concordance with prior studies [7, 14, 24–26].

Our confirmation of myocardial fibrosis in Chagas-seropositive patients, in different stages of disease severity, coincides with previous studies that reported myocardial fibrosis in patients within the indeterminate form [22]. Previous studies have quantified myocardial fibrosis by CMR in Chagas disease patients [16, 26, 27]. Rochitte et al. evaluated CMR with the use of LGE and reported fibrosis in 68.6% of all individuals evaluated and 100% in those with ventricular tachycardia [15]. Noya-Rabelo et al. detected LGE in 64% of patients with Chagas disease [27]. In addition, Regueiro et al. reported fibrosis in 7.4% of patients in the indeterminate form, 15.8% in the cardiac stage without ventricular dysfunction, and 52.4% in those with ventricular dysfunction. It is important to stress that myocardial fibrosis detected within the indeterminate form can be present in such low degrees that do not lead to myocardial dysfunction or remodeling, which requires very sensitive techniques to be detected [22]. In this way, CMR was considered in this study because it has been considered a sensitive technique to detect small myocardial tissue abnormalities in Chagas heart disease, hardly detected by traditional methods, such as echocardiogram, and stands out amongst imaging techniques in the quantification of myocardial fibrosis in this disease [6, 14–16, 18, 22].

We found that patients with LGE had a trend toward worse NYHA functional class compared to patients without LGE, although LGE was found in 25 (52.1%) patients with NYHA functional class I and 22 (45.8%) patients with NYHA functional class II, and it was found in a patient with NYHA functional class III. LVEF and CMR-derived LV volumes showed high negative correlation with echocardiographic measurements for LV telediastolic volume (*r*_*s*_=−0.75), LV telediastolic volume index (*r*_*s*_=−0.77), LV telesystolic volume (*r*_*s*_=−0.90), LV telesystolic volume index (*r*_*s*_=−0.91), LV mass (*r*_*s*_=−0.71), and LV mass index (*r*_*s*_=−0.73). In addition, LVEF showed a high positive correlation with RVEF (*r*_*s*_=−0.69) as shown in Figure 2(b).

Although usually associated with reduced LVEF, we found that RVEF was significantly lower in G3 patients. This finding is in accordance with recent studies which suggest that RV systolic dysfunction in Chagas disease depends mainly on LV increased afterload [28, 29]. Furthermore, the VD telesystolic volume and VD telesystolic volume index were statistically higher in G3 patients (*p* < 0.05). Prominent inflammation, fibrosis, and vasculitis in the right chambers were shown in experimental models of mice infected with *T. cruzi*, even during the acute infection phase [30]. In humans, endomyocardial biopsies on the right side of the interventricular septum revealed histopathologic abnormalities in most patients with chronic Chagas disease [31, 32].

Edema was observed in 8 (9.8%) patients, 2 (5.4%) of G1, 4 (12.1%) of G2, and 2 (16.7%) of G3. It was observed mainly at the basal inferolateral segment in 7 (87.5%) cases. Edema distribution is represented in a 17-segment model in Figure 1(b). All patients with cardiac edema presented LGE, being that it was located more frequently in the subepicardium and mesocardium when compared with nonedema patients (*p* < 0.05). Greater LV telesystolic volume and LV telesystolic volume index were found in those patients (*p* < 0.05).  Table 2 shows the clinical characteristics of patients with cardiac edema.

The presence of edema in the chronic phase of the disease was previously related with severe impairment of myocardial status [22] and may represent the evidence that the inflammation of Chagas disease is maintained for an extended period after the acute phase, as previously reported. Thus, edema finding is probably related to the persistence of an active inflammatory process in the chronic phase.

**Figure 3:**
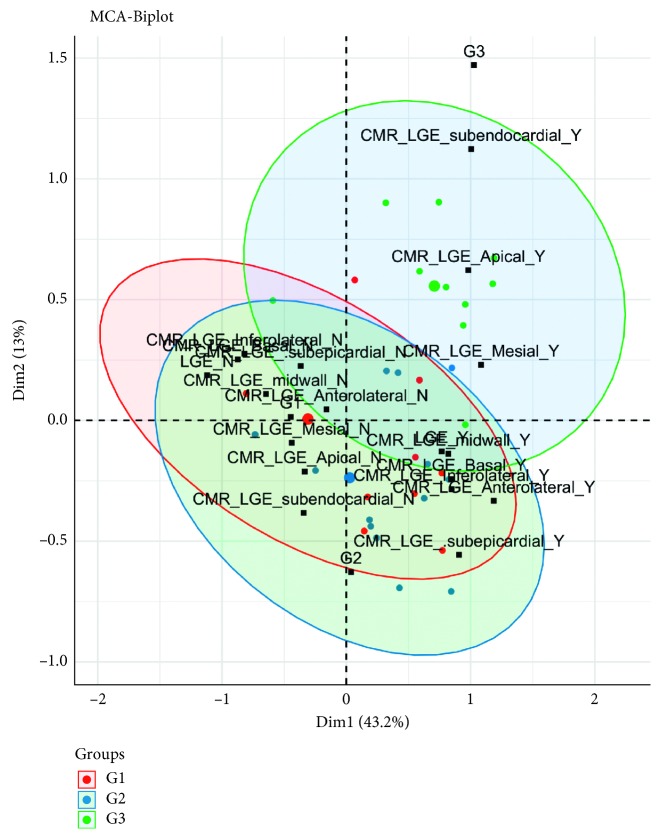
Multiple correspondence analyses of the variables associated with the presence and location of LGE within LVEF groups. It shows the difference between the variables associated with G3 versus G2 and G1 groups.

In Figure 3, it is possible to visualize a structural organization for the variables and categories in a dimensional space, which allows the identification of patterns in the data and associations between the investigated parameters. Although we observed a heterogeneous LGE pattern, as previously reported [26, 27], an association between belonging to G3 and the presence of LGE in the subendocardial segment in apical and mesial locations were found in the MCA analysis, whose first two dimensions accounted for almost 60% of the variability between groups. An association between belonging to G3 and the presence of LGE in the subendocardial segment in apical and mesial locations is easy to perceive in Figure 3. Although it seems that belonging to G2 and presence of LGE in the midwall and subepicardial segments in a basal, inferolateral, and anterolateral locations are associated, this relation is not supported by the analysis. On the contrary, despite being in different quadrants, there is no clear division between the variables associated with G1 and G2 patients.

Our findings indicate that the concept of indeterminate form of Chagas disease, although valid for the collective prognostic evaluation of this group of patients, should be updated in the analysis of individual cases, considering the findings obtained by the application of more accurate diagnostic methods, such as CMR [14, 15, 18, 33–35]. So, even chagasic patients with little or no clinical manifestation of cardiac involvement and performing risk activities or requiring effort should be considered for further careful analysis in order to identify subclinical myocardial damage and risk assessment and prevention of cardiac dysfunction.

## 4. Conclusions

The aim of this study was to characterize CMR findings caused by Chagas at different stages of disease severity in Chagas-seropositive patients and solve the hypothesis that edema could occur in patients with chronic chagasic cardiomyopathy and corresponds to the persistence of the inflammatory process in the myocardium. We found that edema can be found in patients with chagasic cardiomyopathy in the chronic stage. In later stages of cardiac dilation with low LVEF, the LGE pattern involves subendocardium and mid locations. Deteriorations of RV and LV are positively correlated without evidence of fibrosis in the RV.

## Figures and Tables

**Table 1:** Clinical characteristics of Chagas heart disease patients classified by groups.

	Total *n*=82	Group 1FE > 60% *n*=37	Group 260% < FE > 35% *n*=33	Group 3FE < 35% *n*=12	*p* value
Age (years)	58.9 (SD 9.9)	57.6 (SD 11.0)	58.8 (SD 9.1)	63.0 (SD 7.7)	0.13
BMI	26.2 (SD 4.3)	26.7 (SD 3.9)	26.0 (SD 4.3)	25.6 (SD 5.7)	0.38
Socioeconomic	2.0 (SD 1.0)	2.25 (SD 1.0)	1.42 (SD 0.99)	1.4 (SD 0.7)	0.02 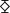
Female (%)	53 (64.6)	30 (81.0)	18 (54.5)	5 (41.7)	0.013 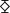
Blood pressure (%)	39 (47.6)	18 (50.0)	12 (40.0)	9 (75.0)	0.12
Type 2 diabetes (%)	7 (8.9)	4 (11.1)	2 (6.7)	1 (8.3)	0.82
DLP (%)	12 (15.4)	8 (22.2)	2 (6.7)	2 (16.7)	0.22
Smoking (%)	3 (3.8)	0 (0)	2 (6.7)	1 (8.33)	0.25
NYHA	1.5 (SD 0.5)	1.4 (SD 0.5)	1.4 (SD 0.6)	1.7 (SD 0.4)	0.19

*CMR*					
LGE (%)	48 (58.5)	16 (43.2)	21 (63.6)	11 (91.7)	0.01❖ 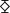
FEVD	54.2 (SD 9.8)	58.6 (SD 5.5)	55.0 (SD 6.8)	38.2 (SD 11.6)	0.01❖ 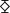
LV telediastolic volume	153.2 (SD 60.5)	123.0 (SD 32.1)	149.0 (SD 39.7)	256 (SD 67.2)	0.01❖ 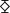 ⦿
LV telediastolic volume index	87.1 (SD 34.7)	70.9 (SD 16.9)	82.5 (SD 24.7)	150.6 (SD 29.8)	0.01❖ 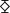
LV telesystolic volume	78.5 (SD 60.1)	43.5 (SD 14.1)	74.5 (SD 33.0)	197.0 (SD 57.0)	0.01❖ 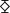 ⦿
LV telesystolic volume index	44.2 (SD 35.8)	24.3 (SD 7.6)	41.0 (SD 20.1)	114.0 (SD 26.5)	0.01❖ 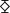 ⦿
LV mass	105.4 (SD 40.6)	87.7 (SD 19.7)	98.5 (SD 25.5)	163.0 (SD 54.7)	0.01❖ 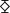
LV mass index	57.9 (SD 21.3)	49.2 (SD 13.4)	53.0 (SD 15.3)	89.7 (SD 20.0)	0.01❖ 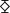
VD telediastolic volume	128.1 (SD 42.4)	122.0 (SD 35.3)	132.0 (SD 41.9)	136.0 (SD 62.1)	0.48
VD telediastolic volume index	72.1 (SD 21.3)	69.5 (SD 18.1)	73.1 (SD 24.1)	77.4 (SD 23.0)	0.51
VD telesystolic volume	58.9 (SD 29.2)	49.8 (SD 16.1)	60.2 (SD 27.0)	83.9 (SD 48.4)	0.01❖ 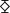
VD telesystolic volume index	33.9 (SD 15.1)	28.5 (SD 8.3)	34.8 (SD 16.0)	48.2 (SD 19.5)	0.01❖ 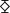

*LGE*					
Transmural (%)	15 (31.2)	3 (18.7)	8 (38.1)	4 (41.6)	0.41
Subendocardic (%)	27 (56.2)	7 (43.7)	10 (47.6)	10 (90.9)	0.03❖
Subepicardic (%)	26 (54.2)	7 (43.7)	14 (66.7)	5 (45.4)	0.31
Midwall (%)	41 (85.4)	12 (75.0)	20 (95.2)	9 (81.8)	0.21
Basal segments^*∗*^ (%)	35 (72.9)	10 (62.5)	18 (85.7)	7 (63.6)	0.21
Apical segments^*∗*^ (%)	21 (43.7)	6 (37.5)	7 (33.3)	8 (72.7)	0.08
Midcavity segments^*∗*^ (%)	18 (37.5)	4 (25.0)	7 (33.3)	7 (63.7)	0.11

*Laboratory*					
Creatinine	0.9 (SD 0.2)	0.84 (SD 0.2)	0.89 (SD 0.2)	1.1 (SD 0.3)	0.00❖
BUN	17.6 (SD 7.9)	16.2 (SD 5.6)	18.5 (SD 8.3)	19.4 (SD 11.6)	0.34

Continuous variables are expressed as mean (SD, standard deviation). Categorical variables are expressed as the number of patients (%). BMI: body mass index; NYHA FC: New York Heart Association functional class. Post hoc comparisons between groups were not statistically significant otherwise indicated ⦿: significant difference between G2 and G1; 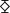: significant difference between G3 and G1; ❖: significant difference between G3 and G2. ^*∗*^Obtained from http://www.pmod.com/files/download/v34/doc/pcardp/3615.htm.

**Table 2:** Clinical characteristics of Chagas heart disease in patients with cardiac edema.

	Edema(*n*=8)	Nonedema(*n*=74)	*p* value
Age	53.8 (SD 12.9)	59.4 (SD 9.5)	0.12
BMI	27.9 (SD 4.3)	26.1 (SD 4.3)	0.25
Socioeconomic	1.75 (SD 0.9)	2.07 (SD 1.0)	0.39
Female (%)	6 (75.0)	47 (63.5)	0.79
Blood pressure (%)	3 (37.5)	36 (51.4)	0.71
Type 2 diabetes (%)	1 (12.5)	11 (15.7)	0.99
DLP (%)	10 (16.9)	6 (24.0)	0.29
Smoking (%)	1 (12.5)	2 (2.9)	0.71
NYHA	1.8 (SD 0.5)	1.4 (SD 0.5)	0.12

*CMR*			
FEVI	45.6 (SD 15.4)	54.6 (SD 14.6)	0.10
LGE (%)	8 (100)	40 (54.1)	0.03^*∗*^
FEVD	51.1 (SD 5.9)	54.5 (SD 10.2)	0.36
LV telediastolic volume	178.0 (SD 78.3)	151.0 (SD 58.3)	0.23
LV telediastolic volume index	104.0 (SD 51.6)	85.3 (SD 32.3)	0.15
LV telesystolic volume	118.0 (SD 79.4)	74.2 (SD 56.7)	0.04^*∗*^
LV telesystolic volume index	69.6 (SD 50.1)	41.4 (SD 31.6)	0.03^*∗*^
VD telediastolic volume	142.0 (SD 23.7)	127.0 (SD 43.9)	0.34
VD telediastolic volume index	81.0 (SD 10.4)	71.1 (SD 22.0)	0.21
VD telesystolic volume	66 (SD 17.2)	58.2 (SD 30.2)	0.47
VD telesystolic volume index	39.0 (SD 7.6)	33.4 (SD 15.6)	0.31

*LGE*			
Transmural (%)	3 (37.5)	12 (16.2)	0.32
Midwall (%)	7 (87.5)	24 (32.4)	0.01^*∗*^
Subendocardic (%)	2 (25.0)	15 (20.3)	0.99
Subepicardic (%)	7 (87.5)	12 (16.2)	0.00^*∗*^
Basal (%)	7 (87.5)	27 (67.5)	0.47
Septal (%)	2 (25.0)	8 (20.0)	0.99
Mesial (%)	1 (12.5)	16 (40.0)	0.28
Apical (%)	4 (50.0)	17 (42.5)	0.99

*Laboratory*			
Creatinine	0.9 (SD 0.2)	0.9 (SD 0.2)	0.93
BUN	20.5 (SD 6.9)	17.2 (SD 8.1)	0.40

Continuous variables are expressed as mean (SD, standard deviation). Categorical variables are expressed as number of patients(%). ^∗^Significant difference between cardiac edema and nonedema patients.

## Data Availability

The data used to support the findings of this study may be released upon application to the Fundación Universitaria de Ciencias de la Salud (FUCS), Bogotá, Colombia, who can be contacted at gestion.conocimiento@fucsalud.edu.co.
